# Qualitative reasoning of dynamic gene regulatory interactions from gene expression data

**DOI:** 10.1186/1471-2164-11-S4-S14

**Published:** 2010-12-02

**Authors:** Yu Chen, Byungkyu Park, Kyungsook Han

**Affiliations:** 1 School of Computer Science and Engineering, Inha University, Incheon, Korea

## Abstract

**Background:**

A gene regulatory relation often changes over time rather than being constant. But many gene regulatory networks available in databases or literatures are static in the sense that they are either snapshots of gene regulatory relations at a time point or union of successive gene regulations over time. Such static networks cannot represent temporal aspects of gene regulatory interactions such as the order of gene regulations or the pace of gene regulations.

**Results:**

We developed a new qualitative method for representing dynamic gene regulatory relations and algorithms for identifying dynamic gene regulations from the time-series gene expression data using two types of scores. The identified gene regulatory interactions and their temporal properties are visualized as a gene regulatory network. All the algorithms have been implemented in a program called GeneNetFinder (http://wilab.inha.ac.kr/genenetfinder/) and tested on several gene expression data.

**Conclusions:**

The dynamic nature of dynamic gene regulatory interactions can be inferred and represented qualitatively without deriving a set of differential equations describing the interactions. The approach and the program developed in our study would be useful for identifying dynamic gene regulatory interactions from the large amount of gene expression data available and for analyzing the interactions.

## Background

Many mechanisms of biological processes are controlled by complex regulatory interactions between genes rather than by a single gene [1]. Therefore, identifying the gene regulatory interactions is essential to improving our understanding of biological processes. A gene regulatory relation often changes over time rather than being constant. However, many gene regulatory networks available in databases or literatures are static in the sense that they are either snapshots of gene regulatory relations at a time point or union of successive gene regulations over time. Static gene regulatory networks are simpler and easier to construct and understand than dynamic networks, but temporal aspects of gene regulations such as the order of the gene regulatory interactions and the pace of the interactions are ignored in static networks.

A gene involved in regulatory interactions with others has at least one activator or inhibitor. An activator initiates the transcription of the gene, so high level expression of the gene is not possible without an activator [2]. Thus, identifying genes and their activators or inhibitors is the key to constructing gene regulatory networks. Silvescu et al. [1] characterize the gene regulatory network in a Boolean model with multiple-time delays. But the Boolean model is restricted to logical relationships between variables. Probabilistic Boolean networks [3] and dynamic Bayesian networks [4] can reconstruct longitudinal regulatory networks from a set of mathematical equations if the equations precisely specify the networks, but fail when the underlying model is not correct [5].

In general dynamic relations are best represented by a system of differential equations, but differential equations are not typically used to represent dynamic gene regulatory relations. This is because dynamic gene regulatory interactions are not understood fully enough to derive differential equations despite the large amount of gene expression data available today. Even if differential equations are derived, they are often hard to solve. As shown later in this paper, we have developed a qualitative method for inferring dynamic gene regulatory interactions and visualizing them without deriving or solving a set of differential equations.

This paper presents a computational approach to uncovering gene regulatory relations and their temporal properties from a time-series gene expression data using a modified Pearson correlation coefficient and a new score scheme. For the temporal properties of gene regulatory relations, we infer the order of the gene regulatory interactions and the pace of the interactions. The identified gene regulatory interactions and their temporal aspects are stored in the regulation list and visualized as a gene regulatory network. All the algorithms have been implemented as a program called GeneNetFinder (http://wilab.inha.ac.kr/genenetfinder/) and tested on several gene expression data. The rest of this paper presents the algorithms and their experimental results.

## Methods

### Scoring scheme for gene regulatory relationships

**Figure 1:**
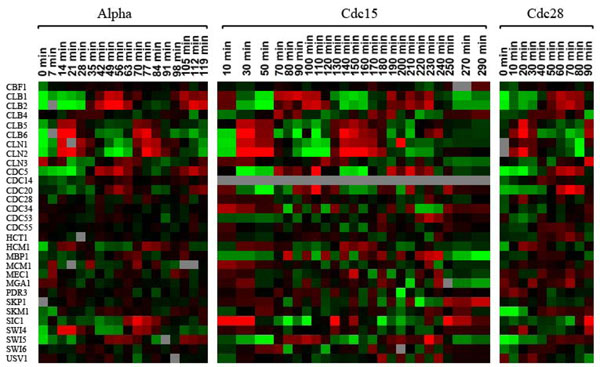
**Gene expression of 30 genes during the yeast cell cycle**[6]**.** Each row represents a gene and each column represents a time point. Red areas indicate an increase in mRNA abundance while green indicates a decrease in mRNA abundance with respect to the control.

The gene expression data of *m* genes with *n* samples is represented as an *m* × *n* matrix, where rows represent genes and columns represent various samples such as experimental conditions or time points in a biological process. Each element of the matrix represents the expression level of a particular gene in a particular sample. Two genes with similar expression patterns tend to be co-expressed at different time points. Figure 1 shows an example of the gene expression data for yeast genes during the yeast cell cycle, obtained from the Yeast Cell Cycle Analysis Project [6].

The gene expression matrix is analyzed for similarity between gene expressions at different time points. Three metrics are often used to measure the similarity of genes: Pearson correlation coefficient [7], Euclidean distance [8] and Spearman correlation [9]. To evaluate the regulatory relation between two genes, we modified the Pearson correlation coefficient. *R*1(*X,Y*, *i, p*) in Equation 1 represents the correlation between gene *X* at time point *i* and gene *Y* at time point *i + p. p* is the time span of the gene regulation.(1)

In Equation 1, *N* is the total number of time points contained in the time span, *X_k_* and *Y_k_* are the expression levels of genes *X* and *Y* at time *k,* and  and  are the average gene expression levels at all time points of the time span. The R1 score is in the range of [-1, 1]. Among the total *i × p* candidate regulations, the regulation with the maximum absolute value of *R*1(*X, Y, i, p*) is selected as the regulatory relation between genes *X* and *Y.* If the expression level of gene *X* increases before that of *Y* increases, *X* is a candidate activator of gene *Y;* if the expression level of gene *X* increases before that of *Y* decreases, *X* is a candidate inhibitor of *Y.*

**Figure 2:**
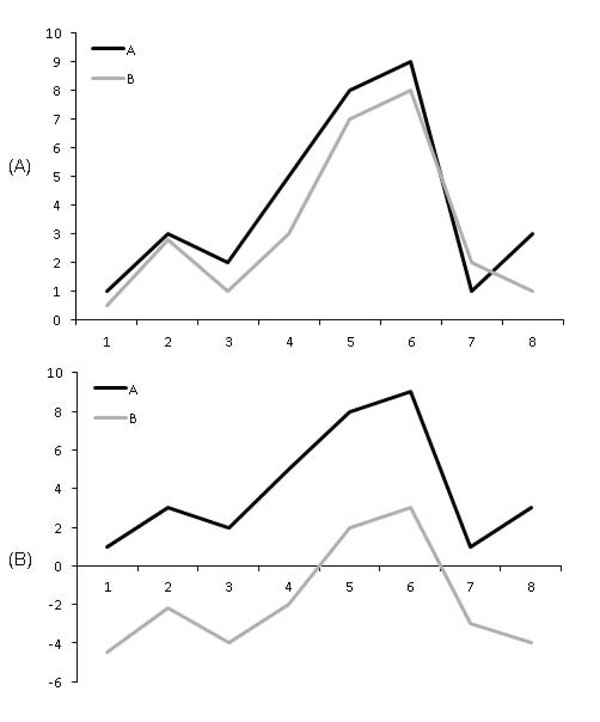
**Example of the gene regulatory relations with the same R1 score but different expression levels.** The regulatory relations of two genes have the same R1 score of 0.94 when *p =* 0, but have different expression levels in (A) and (B).

The modified Pearson correlation coefficient *R*1 is useful for finding gene regulatory relations with a significant change in expression levels. But, it cannot distinguish gene regulatory relations with the same correlation but different gene expression levels (see Figure 2 for an example). Therefore, we use an additional score *R*2*,* which is the Euclidean distance of the expression levels of the two genes. The score *R*2 for the gene regulatory relation between *X* and *Y* is computed by Equation 2.(2)

where

In Equation 2,  and  are the average gene expression levels at all time points in the time span. *X_max_* is the maximum gene expression value of gene *X*.  and  are the maximum and minimum gene expression value of gene *Y,* respectively.

Both *R*1 and *R*2 scores are intended to represent a relation between a regulator gene X and its potential receiver gene Y. The regulatory relation between X and Y is not symmetric, so *R*1(*X, Y*) *≠ R*1(*Y, X*) and *R*2(*X,Y*) *≠ R*2(*Y,X*)*.* An interesting observation from the actual data is that two genes with *R*2 score < 3 tend to have an inductive relation, whereas those with *R*2 scores > 6 tend to have an inhibitory relation. For example, in the dataset of 30 yeast genes, 89.2% of the activations have *R*2 scores < 3, and 91.4% of the inhibitions have *R*2 scores > 6. In an extended dataset of 6,177 yeast genes, 80.4% of the activations have *R*2 scores < 3, and 92.1% of the inhibitions have *R*2 scores > 6. Hence, we consider a gene regulation with *R*2 score < 3 as an activation, and that with score > 6 as an inhibition.

### Inferring gene regulatory relationships

In the microarray data for gene expression, we use the log-ratio (in base 2) of the red and green intensities. Thus, genes with mRNA abundance have positive log-ratios whereas those with absence of mRNA have negative log-ratios. From the gene expression profiles, we identify the gene regulations and include them in the regulation list. In the regulation list, +A(t) indicates that gene A is up-regulated at time t, and -A(t) indicates that gene A is down-regulated at time t. The symbol '→' represents a directional relationship between genes. There are four possible gene regulatory relations between two genes:

1. +A(*t*_1_) → +B(*t*_2_): up-regulation of A at time *t*_1_ is followed by up-regulation of B at time *t*_2_ (*t*_2_ > *t*_1_).

2. -A(*t*_1_) *→* +B(*t*_2_): down-regulation of A at time *t*_1_ is followed by up-regulation of B at time *t*_2_ (*t*_2_ > *t*_1_).

3. +A(*t*_1_) -*>* -B(*t*_2_): up-regulation of A at time *t*_1_ is followed by down-regulation of B at time *t*_2_ (*t*_2_ > *t*_1_).

4. -A(*t*_1_) *→* -B(*t*_2_): down-regulation of A at time *t*_1_ is followed by down-regulation of B at time *t*_2_ (*t*_2_ > *t*_1_)*.*

The regulatory relation of gene A with gene B is determined by the sign of the *R*1 score of the genes. A relation with a positive *R*1 score implies that gene A activates gene B whereas a regulation with a negative *R*1 score implies that A inhibits B. The *R*1 score of each gene regulation is iteratively calculated using Algorithm 1. For genes *A* and *B,* the regulation with the largest absolute R1 score is chosen for the regulation between the genes and represented as *R*1(*A, B, t*_1_, *p*). Algorithm 1 provides the top-level description of the method for inferring gene regulations and constructing a list with the regulations.

#### Algorithm 1

Construct a regulation list

1: Compute *R*1(*A, B, t*_1_, *p*) between gene A at time point *t*_1_ and gene B at time point *t*_1_ + *p* for all pairs of genes.

2: Select the regulation with the largest absolute value of *R*1(*A, B, t*_1_, *p*)*.*

3: If 0 <*p <* 6, classify the regulation into one of the four types, +A(*t*_1_) *→ +*B(*t*_1_*+p*)*, -*A(*t*_1_ ) *→ +*B(*t*_1_*+p*)*,* +A(*t*_1_) → -B(*t*_1_*+p*)*,* -A(*t*_1_) *→ -*B(*t*_1_*+p*)*,* and add it to the regulation list.

4: If *p =* 0, two genes are co-expressed or co-inhibited, and such gene regulation is not added to the regulation list.

5: If the new gene regulation is already in the regulation list, merge it with the previous regulation.

6: Go to step 2 to find the next gene regulation until no more regulation found.

**Figure 3:**
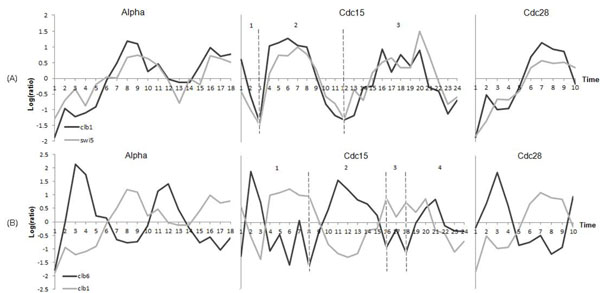
**Changes in gene expression levels over time spans.** (A) Gene expression levels of swi5 and clb1 in 3 time spans labeled as Alpha, Cdc15 and Cdc28. The label of a time span indicates an inducer of the time span. The time span Cdc15 is split into 3 sub-timespans, separated by dashed line. (B) Gene expression levels of clb6 and clb1 in 3 time spans of Alpha, Cdc15 and Cdc28. The label of a time span indicates an inducer of the time span. The time span Cdc15 is split into 4 sub-timespans, separated by dashed line.

After we construct a regulation list, we compute the *R*2 score for the gene pairs in the regulation list. Some local maximum or minimum values are ignored when computing the *R*2 score in a long time span. For example, all the three time spans shown in Figure 3A include at least 10 time points (18 time points in alpha-factor, 24 in cdc15, and 10 in cdc28). Expression levels of gene CLB1 has several wave peaks in the time span of CDC15, but only the maximum value in sub-timespan 2 of CDC15 will be selected when computing the *R*2 score in the time span of CDC15. Time spans are divided into smaller sub-timespans as follows, and the *R*2 score is not computed for sub-timespans with less than 6 time points.

1. A time point with the minimum expression level of the regulator gene becomes a splitting point of the time span.

2. Each sub-timespan starts with at least 3 consecutive time points that have a positive slope of a curve representing gene expression levels, and ends with at least 3 consecutive time points with a negative slope.

3. Each sub-timespan encompasses at least 6 time points, including the start and ending time points.

For example, the time span CDC 15 of Figure 3A is the longest one in the gene regulatory relation +CLB1(T)-> +SWI5(T+1), and split into 3 sub-timespans. The first sub-timespan of CDC15 has less than 6 time points, so the *R*2 score is not computed for the first sub-timespan. The *R*2 scores for the second and third sub-timespans are 0.24 and 0.38, respectively. Figure 3B is another example of a gene regulatory relation +CLB6(T) -> -CLB1(T+1). The time span CDC15 is split into 4 sub-timespans, and the third sub-timespan has only 3 time points. So, the *R*2 score is computed for the remaining three sub-timespans, which are 27.02, 20.34, and 9.87, respectively.

### Visualization of gene regulatory networks

All gene regulations identified in the previous step are visualized as a 2-dimensional gene regulatory network, in which a node represents a gene. Edge types and edge labels of the network represent gene regulatory relations. Arrows represent inductive interactions (relations+A(t1) -> +B(t2) and-A(t1) -> +B(t2)) and blocked arrows represent inhibitory interactions (relations +A(t1) -> -B(t2) and -A(t1) -> -B(t2)). The regulator gene, type of regulation (+ for induction and - for inhibition), and time delay of the regulation are annotated as edge labels. Each edge is labeled with *R/s/T* to indicate a regulator gene *R,* sign *s* of the log-ratio of the expression level of *R,* and the time delay *T* of the regulation.

**Figure 4:**
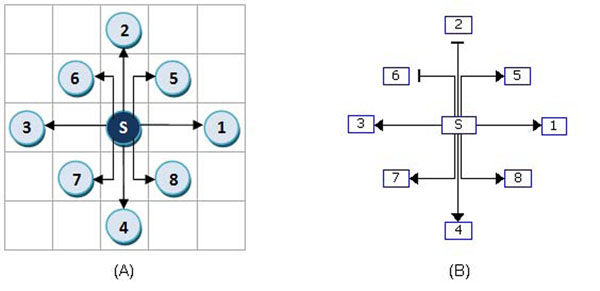
**Example of the grid layout.** (A) Node S with the highest degree is placed in the center grid, and the nodes connected to S are placed in the adjacent grids in the specified order. (B) Grid layout by GeneNetFinder.

For visualization of gene regulatory networks, two layout algorithms have been developed: grid layout and layered layout. As described in Algorithm 2, the grid layout algorithm positions all nodes at grid points. The node with the highest degree will be placed at the center grid point (node S in Figure 4A). Then, we position all nodes connected to the center node at adjacent grid points. Nodes with a higher degree are positioned earlier than those with a lower degree in the east, north, west, south, northeast, southwest, and southeast grid point of the current node (nodes 1-8 of Figure 4). Other nodes connected to the positioned nodes are placed in the same manner.

The layered layout algorithm, described in Algorithm 3, puts all nodes to horizontal layers. The node with the maximum degree is assigned to the top layer, and the nodes connected to the node are put in the next layer. If a layer has two nodes connected to each other, it makes a new layer above the layer and moves the node with a smaller degree to the new layer. The layered layout usually takes more time than the grid layout.

#### Algorithm 2

Grid layout

1: Find the node with the highest degree, and place it at the center grid point.

2: If there is a tie, select a node with a higher out-degree. Position all nodes connected to the center node in the adjacent grids. Nodes with a higher degree are positioned earlier than those with a lower degree in the east, north, west, south, northeast, northwest, southwest, and south east grid of the center node. If more than 8 nodes are connected to the center node, put the 9th node to the east of the 1st node, the 10th node to the north of the 2nd node, and so on.

3: Repeat step 2 for nodes connected to nodes that are already positioned. If an adjacent grid is occupied, move to the next possible position until it is available.

4: If there are disconnected nodes, repeat step 1 for the nodes and put them to the right of the previous subgraph.

#### Algorithm 3

Layered layout

1: Put the node with the maximum degree at layer 1. If there is a tie, select a node with a higher out-degree.

2: Assign the nodes connected the nodes at layer i to layer i+1.

3: Repeat steps 1 and 2 for the remaining nodes.

4: If two nodes at the same layer are connected to each other, make a new layer between the layer and the upper layer and move the node with a smaller degree to the new layer. Nodes with 0 out-degrees are also moved to the new layer.

5: Order the nodes in each layer by the Barycenter method [10].

6: If there are disconnected nodes, repeat steps 1-5 for the nodes and put them to the right of the previous subgraph.

## Results and discussion

We implemented the algorithms in a program called GeneNetFinder using Microsoft Visual *C#.* GeneNetFinder is executable on any Windows systems, and the program and sample data of GeneNetFinder are available at http://wilab.inha.ac.kr/GeneNetFinder. Given a time-series data of gene expressions in log-ratios, it identifies gene regulatory interactions and visualizes them. This section shows the experimental results with the gene expression data of yeast cell cycling and human cell cycling.

### Microarray data of 30 genes in the yeast cell cycle

The dataset of the yeast cell cycle, shown in Figure 1, includes 30 genes of yeast cell cycle from the *Saccharomyces cerevisiae* cell cultures [6]. The 30 yeast genes are known to be involved in the cell-cycle regulations. For the cell cycle genes, we first selected well-known genes and their regulator or regulated genes. CLB genes, for example, are known to promote cell cycle progression into mitosis [11]. CLN genes were selected because they have regulatory relations with CLB genes. The remaining genes were chosen randomly. There are 18, 24, 10 time points in the time spans of alpha-factor, cdc15 and cdc28, respectively.

From the dataset, GeneNetFinder identified 73 gene regulations and constructed a list of the gene regulations (Table 1). Figure 3A shows the gene expression levels of CLB1 and SWI5. Gene SWI5 encodes a transcription factor that activates transcription of genes expressed at the M/G1 boundary and in G1 phase. Genes CLB1 and SWI5 showed similar expression patterns and the R1 score computed for their relation was 0.85. For the two genes, GeneNetFinder inferred a gene regulatory relation +CLB1(T) → +SWI5(T+1), which means that up-regulation of CLB1 is followed by up-regulation of SWI5 at the next time point. This regulatory relation agrees with the experimental results by Althoefer [12]. The time span CDC15 of Figure 3A is the longest on in the gene regulatory relation +CLB1(T) →- +SWI5(T+1), and divided into 3 sub-sections. The 3 sub-sections showed the *R*2 scores of 0.53, 0.24 and 0.38. Figure 3B shows another gene regulatory relation +CLB6(T) *→* -CLB1(T+ 1). This relation also had the time span of CDC15, which was split into 3 sub-timespans with the *R*2 scores of 27.02, 20.34 and 9.87.

**Table 1:** Gene regulations identified in the time-series expression data of yeast cell cycle.

Genes	T	T+1	T+2	T+3
CLB1	+CLB1(T)→ -CLN2(T+1)			
	+CLB1(T) → +SWI5(T+1)	+CLB1(T+1) → +CDC20(T+3)	+CLB1(T+2) → +CDC20(T+4)	
	-CLB1(T) → -CDC20(T+1)			
CLB2		*+ CLB2(T+1) → +SICl(T+3)*	+CLB2(T+2) → +SWI4(T+5)	+CLB2(T+3) → +SWI4(T+6)
CLB6	+CLB6(T)→ -CLB1(T+1)	+CLB6(T+1) → -CLB1(T+2)	+CLB6(T+2)→ -CLB1(T+3)	
	+CLB6(T) → -CLB2(T+1)	+CLB6(T+1) → -CLB2(T+2)	+CLB6(T+2)→ -CLB2(T+3)	
CLN1	-CLN1(T) → -CLB4(T+1)	-CLN1(T+1) → -CLB4(T+2)		
CLN2	-CLN2(T) → -SWI6(T+1)		-CLN2(T+2) → +SIC1(T+5)	
CLN3	+CLN3(T) → +SIC1(T+1)			
	+CLN3(T) → +CLB6(T+3)			
CDC5	+CDC5(T) → +CDC14(T+1)	+CDC5(T+1) → +CDC14(T+2)		+CDC5(T+3) → +CDC20(T+5)
	+CDC5(T) → +CDC20(T+1)	+CDC5(T+1) → +CDC20(T+2)		
CDC14	-CDC14(T) → +CLN1(T+1)	-CDC14(T+1) → +CLN1(T+2)	-CDC14(T+2) → +CLN1(T+3)	
CDC28	+CDC28(T) → +SWI4(T+1)			
CDC34			-CDC34(T+2) → +CDC34(T+5)	
CDC53	*+ CDC53(T) → -CLN3(T+1)*			
CDC55	+CDC55(T) → +USV1(T+1)	+CDC55(T+1) → +USV1(T+2)	+CDC55(T+2) → +USV1(T+3)	+CDC55(T+3) → +USV1(T+5)
	+CDC55(T) → +SWI5(T+1)	+CDC55(T+1) → +SWI5(T+2)	+CDC55(T+2) → +SWI5(T+3)
HCM1	+HCM1(T) → -CLB5(T+1)		+HCM1(T+2) → -CLB5(T+4)	
	+HCM1(T) → -CLN1(T+1)			
MCM1		+MCM(T+1) → -MBP1(T+2)	+MCM(T+2)→ -MBP1(T+4)	
MEC1	-MEC1(T) → +CBF1(T+1)			-MEC1(T+3) → +CBF1(T+5)
MGA1	+MGA1(T) → +CDC5(T+1)			
PDR3	+PDR3(T) → +SWI5(T+1)	+PDR3(T+1) → +SWI5(T+2)	+PDR3(T+2) → +SWI5(T+3)	
SKP1	-SKP1(T) → -SWI4(T+1)			-SKP1(T+3) → -MBP1(T+4)
SKM1	-SKM1(T) → +CLB6(T+1)	-SKM1(T+1) → +CLB6(T+2)	-SKM1(T+2) → +CLB6(T+3)	-SKM1(T+3) → +CLB6(T+4)
SIC1		*-SIC1(T+1) → +SWI5(T+3)*	*-SIC 1(T+2) → +SWI5(T+4)*	*-SIC 1(T+3) → +SWI5(T+5)*
		*-SIC1(T+1) → -CLN2(T+3)*	*-SIC 1(T+2) → -CLN2(T+4)*	*-SIC 1(T+3) → -CLN2(T+5)*
SWI5	*+SWI5(T) → -CLNl(T+1)*	*+SWI5(T+1) → -CLN1(T+2)*		*+SWI5(T+3)→ -CLN1(T+4)*
	-SWI5(T) → +CLB6(T+1)			
SWI6		-SWI6(T+1) → -SKP1(T+2)		*-SWI6(T+3) → -CDC20(T+6)*
USV1	+USV1(T)→ -SKM1(T+1)	+USV1(T+1)→ -SKM1(T+3)		

Underlined entries denote the regulations determined by experimental methods, and italicized entries denote the regulations implied by previous studies.

There are several resources that can be used to assess the gene regulations inferred by the program. We searched the KEGG, SGD and CYGD databases and literatures to see whether the databases contain a gene regulation that agrees with the identified gene regulation. KEGG has 29,471 pathways whereas SGD (http://www.yeastgenome.org/) and CYPD (http://mips.gsf.de/genre/proj/yeast/) provide genetics and functional networks of the budding yeast *Saccharomyces cerevisiae,* respectively. Gene ontology [13] can also be used to obtain gene regulatory relations; if a gene has a GO annotation of 'regulates' or 'regulated by', the gene can be considered as being involved in a gene regulation.

Gene regulations identified by the program are classified as *certain, possible* and *uncertain* depending on the number of supporting evidences (agreement with the databases, agreement with GO annotations, *R*2 score < 3 for activation and *R*2 score > 6 for inhibition):

1. A gene regulation is certain when it has at least two supporting evidences.

2. A gene regulation is possible when its *R*2 score is either < 3 or > 6, and no other evidences.

3. A gene regulation is uncertain when it has no supporting evidence.

In the dataset of the yeast cell cycle, a total of 73 gene regulations were inferred by GeneNetFinder (Table 1). There were 48 certain gene regulations. 4 out of the 73 gene regulations, +CDC28->+SWI4(T+1),+HCM1(T) -> -CLB5(T+1), +HCM1 -> -CLN1(T+1) and +MGA1(T) -> +CDC5(T+1), had supporting evidences both in the databases and GO annotations. 43 out of the 73 gene regulations showed exact agreement with the known regulations in the databases. Only one gene regulation +USV1(T)->-SKM1(T+1) had the *R*2 score > 6 and the GO annotation about the regulation. These 48 gene regulations are marked as underlined entries in Table 1, and more details are available in Additional file 1.

12 out of the 73 gene regulations, written in italics in Table 1, were possible regulations. They had the *R*2 score either < 3 or > 6 with no further supporting evidence. These regulations should be verified by experimental evidence. The remaining 13 gene regulations had the *R*2 score between 3 and 6 and no supporting evidence, so they are uncertain regulations. Even if the 13 regulations are false positives, at least 82.2% (60 out of 73) of the regulations inferred by GeneNetFinder agreed with known gene regulations. Table 1 shows the regulations for the first four time points only, and there are two more regulations identified at time points T+4 and T+5: -CLB6(T+4) -> +CDC28(T+5) [14] and -CDC20(T+4) -> -CLB6(T+5) [15].

**Figure 5:**
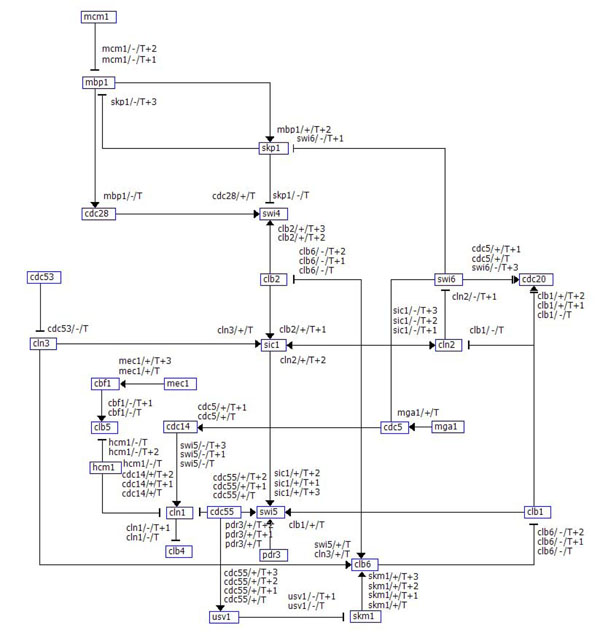
**Visualization of the gene regulatory network of the yeast cell cycle.** The gene regulations of Table 1 are displayed by the grid layout algorithm. Edge types and edge labels represent gene regulatory relations. Arrows represent inductive interactions (relations +A(t1) -> +B(t2) and -A(t1) -> +B(t2)) and blocked arrows inhibitory interactions (relations +A(t1) -> -B(t2) and -A(t1) -> -B(t2)). Each edge is labeled with *R/s/T* to indicate a regulator gene *R,* sign *s* of the expression level of *R,* and the time point *T* of the regulation.

The gene regulations should be consistent with the phase characteristics of the cell cycle. For example, CLB6 promotes progression of cells into the S phase and expresses periodically throughout the cell cycle [16]. CLB1 and CLB2 both promote cell cycle progression into mitosis and their transcripts accumulate during G2 and M. These biological processes are characterized by two regulations, +CLB6 -> -CLB1 and +CLB6 -> -CLB2, and these are included in the regulation list found by GeneNetFinder (Table 1). Figure 5 shows the gene regulatory network of all the gene regulations of Table 1.

### Microarray data of 20 genes in the human cell cycle

**Figure 6:**
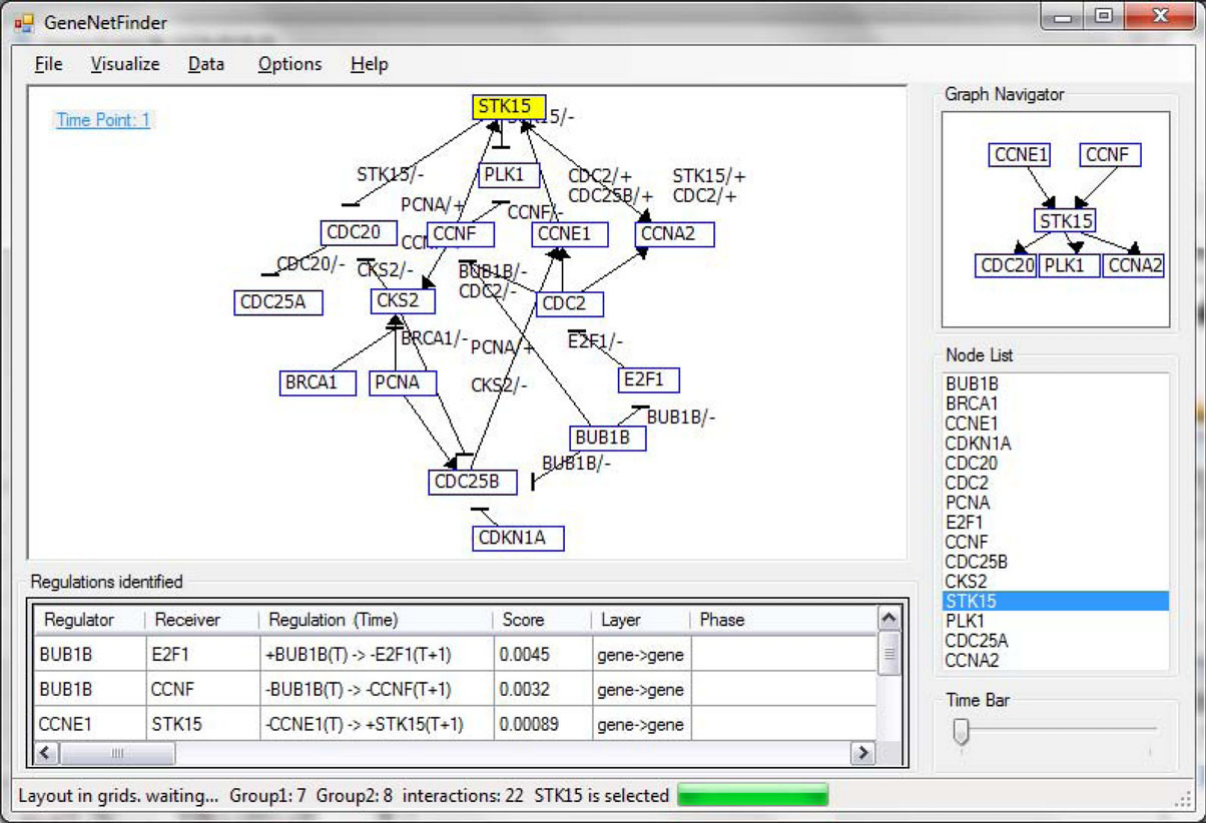
**User interface of GeneNetFinder and the gene regulatory network of the human cell cycle.** The network in the top left corner shows 22 regulations between 15 human genes in the first time period of the human cell cycle, visualized as a layered graph. A list of gene regulations identified by the program are shown below the network. When the user clicks a node in the network, all connected nodes are shown in the graph navigator. The node list shows all the nodes in the network.

This data set includes 20 genes in the human cell cycle [17]. The gene expression during the human cell cycle is synchronized by double thymidine blocking (Thy-Thy1, Thy-Thy2, and Thy-Thy3), thymidine-nocodazole blocking (Thy-Noc) and Mitotic selection (M). Additional file 2 shows the data of 20 genes in the human cell cycle. All regulations identified by GeneNetFinder are given in Additional file 2. 71 of 113 potential gene regulations were found in at least one of KEGG, Entrez Gene, BIND and PUBMED, and 44 of the 71 regulations had been determined by experimental methods. Thus, at least 62.8% of the identified regulations are in agreement with known regulations. Figure 6 shows the regulatory network of 15 human genes in the first time span along with the user interface of GeneNetFinder.

For further evaluation, we selected some genes. Gene CCNA2 encodes proteins of the highly conserved cyclin family which plays an important role in the cell cycle. CCNA2 binds and activates CDC2 kinases, and thus promotes both cell cycle G1/S and G2/M transitions. Then CDC2 encodes proteins which are members of the Ser/Thr protein kinase family. This protein is a subunit of the highly conserved protein kinase complex and essential for G1/S and G2/M phase transitions. In the KEGG pathway database, we found that CDC2 interacts with E2F1 and SKT15, and E2F1 has direct regulatory relationships with CDC2, CCNA2 and BRCA1. The protein encoded by the gene E2F1 is a member of the E2F family of transcription factors. The E2F family plays a crucial role in the control of cell cycle and action of tumor suppressor proteins. In summary, CCNA2 interacts with E2F1, CDC2, and CDKN1A; CDC2 interacts with E2F1, CCNA2, CCNB1, CDC25A, CDC25B, CDC25C and CDKN1A; and CCNB1 interacts with CDC2, CCNF, BRCA1 and CDKN1A. All these regulations agree with the regulations identified by GeneNetFinder.

## Discussion

We tested GeneNetFinder on datasets of different sizes to see the effect of changes in genes on the prediction performance of the program. In the dataset of 90 yeast genes, it inferred 470 gene regulations, and 241 out of the 470 regulations have at least two supporting evidences such as agreement with the known data of databases, agreement with GO annotations, R2 score < 3 for activation, and R2 score > 6 for inhibition (Additional file 3). These regulations are classified as certain regulations in our study. 121 out of the 470 gene regulations have R2 score either < 3 or > 6, and these regulations are possible regulations. Thus, 77.02% of the gene regulations inferred by GeneNetFinder can be considered correct (Table 2). In a similar way, we tested GeneNetFinder on datasets of different number of genes, which were selected randomly from a pool of 90 genes in each run. The prediction was computed by taking the average of 10 runs in each dataset. Datasets of different sizes resulted in slightly different but similar prediction accuracies, and all of them are above 74%. Details are available at http://wilab.inha.ac.kr/genenetfinder/supplements_3.htm.

**Table 2:** Effect of changes in genes on the prediction performance.

#genes in a dataset	20	30	40	50	60	70	80	90
#certain regulations (a)	50	71	114	133	177	221	235	241
#possible regulations (b)	20	33	61	64	79	109	116	121
#total regulations (c)	91	137	227	265	332	426	453	470
Accuracy (%) ((a+b)/c)	76.9	75.9	77.1	74.3	77.1	77.5	77.5	77.0

GeneNetFinder was tested on a different number of genes, which were selected randomly in each run from a pool of 90 yeast genes. The accuracy shown in the table is the average of 10 independent runs. Correct regulations include certain regulations and possible regulations.

There are a few programs that can infer gene regulatory interactions from time-series gene expression data [18,19]. ASIAN [20], for example, finds correlation relationships between gene clusters and visualizes them as an undirected graph. While gene regulatory networks visualized by GeneNetFinder represent gene regulatory interactions between individual genes along with temporal aspects of the interactions, networks visualized by ASIAN represent correlations between gene clusters. Thus, it cannot show regulatory interactions between individual genes nor the order or pace of the interactions. BioTapestry [21] is an interactive tool for building and visualizing gene regulatory networks. For visualizing gene regulatory networks BioTapestry uses different layout methods from GeneNetFinder, and temporal aspects of gene regulatory interactions are not automatically shown as edge labels of the networks. BioTapestry allows flexible edge labels, which can be annotated with any properties specified by the user.

## Conclusions

Gene regulatory interactions usually change over time rather than being constant, but many databases or literatures provide static gene regulatory networks only. They are either snapshots of gene regulatory relations at a time point or union of successive gene regulations over time. Static gene regulatory networks are easier to construct and understand than dynamic networks, but cannot provide information on temporal aspects of gene regulations.

This article has presented an algorithm for qualitatively reasoning dynamic gene regulatory relations from gene expression data using two types of scores, *R*1 and *R*2*.* The algorithm has been implemented in a program called GeneNetFinder. From the time-series data of gene expression, GeneNetFinder infers not only gene regulatory interactions but also the temporal aspects of the regulatory interactions. As for the temporal aspect of gene regulatory relations, it identifies the order of the gene regulatory relations and the pace of the relations. The identified gene regulatory interactions and their temporal aspects are stored in the regulation list and visualized as a gene regulatory network. In the network visualized, gene regulations and their temporal aspects are represented by edge types and edge labels.

We tested GeneNetFinder on several datasets, including the yeast cell cycle data and the human cell cycle data. Experimental results indicate that the dynamic nature of dynamic gene regulatory interactions can be identified and represented qualitatively without deriving or solving a set of differential equations describing the interactions. GeneNetFinder is yet a prototype, but the approach of our work would be useful for identifying dynamic gene regulatory interactions from the large amount of gene expression data available at the present time. In particular, the gene regulatory networks constructed by GeneNetFinder can be used to find new gene regulatory relations or to refine known regulatory relations.

## Competing interests

The authors declare that they have no competing interests.

## Authors contributions

Yu Chen developed the algorithm and prepared the first draft of the manuscript. Byungkyu Park helped Yu Chen develop the method. Kyungsook Han supervised the work and rewrote the manuscript.

## Supplementary Material

Additional file 1 - Regulation of 30 yeast genes during the cell cycle Supplementary data at http://wilab.inha.ac.kr/GeneNetFinder/supplements_1.htm

Additional file 2 - Regulation of 20 human genes during the cell cycle Supplementary data at http://wilab.inha.ac.kr/GeneNetFinder/supplements_2.htm

Additional file 3 - Regulation of 90 yeast genes during the cell cycle Supplementary data at http://wilab.inha.ac.kr/GeneNetFinder/supplements_3.htm.
